# Human cerebral organoids establish subcortical projections in the mouse brain after transplantation

**DOI:** 10.1038/s41380-020-00910-4

**Published:** 2020-10-13

**Authors:** Xin Dong, Shi-Bo Xu, Xin Chen, Mengdan Tao, Xiao-Yan Tang, Kai-Heng Fang, Min Xu, Yufeng Pan, Yuejun Chen, Shuijin He, Yan Liu

**Affiliations:** 1 Institute for Stem Cell and Neural Regeneration, School of Pharmacy, State Key Laboratory of Reproductive Medicine, Nanjing Medical University, Nanjing, 211166 China; 2 Beijing Key Laboratory of Neural Regeneration and Repair, School of Basic Medical Sciences, Advanced Innovation Centre for Human Brain Protection, Capital Medical University, Beijing, 100069 China; 3 School of Life Science and Technology, ShanghaiTech University, Shanghai, 201210 China; 4 The Key Laboratory of Developmental Genes and Human Disease, School of Life Science and Technology, Southeast University, Nanjing, 210096 China; 5 Institute of Neuroscience, CAS Key Laboratory of Primate Neurobiology, CAS Center for Excellence in Brain Science and Intelligence Technology, Chinese Academy of Sciences, Shanghai, 200031 China; 6 Co-innovation Center of Neuroregeneration, Nantong University, Nantong, 226019 China

**Keywords:** Stem cells, Neuroscience, Cell biology

## Abstract

Numerous studies have used human pluripotent stem cell-derived cerebral organoids to elucidate the mystery of human brain development and model neurological diseases in vitro, but the potential for grafted organoid-based therapy in vivo remains unknown. Here, we optimized a culturing protocol capable of efficiently generating small human cerebral organoids. After transplantation into the mouse medial prefrontal cortex, the grafted human cerebral organoids survived and extended projections over 4.5 mm in length to basal brain regions within 1 month. The transplanted cerebral organoids generated human glutamatergic neurons that acquired electrophysiological maturity in the mouse brain. Importantly, the grafted human cerebral organoids functionally integrated into pre-existing neural circuits by forming bidirectional synaptic connections with the mouse host neurons. Furthermore, compared to control mice, the mice transplanted with cerebral organoids showed an increase in freezing time in response to auditory conditioned stimuli, suggesting the potentiation of the startle fear response. Our study showed that subcortical projections can be established by microtransplantation and may provide crucial insights into the therapeutic potential of human cerebral organoids for neurological diseases.

## Introduction

Human cerebral organoids generated from human embryonic stem cells (hESCs) and human induced pluripotent stem cells (hiPSCs) have enormous potential for the study of human brain development and neurological diseases [1–3]. For example, three-dimensional organoids have been used as a model to study the mechanism of ZIKA infection and potential therapeutic drugs [4]. Many groups have studied neural developmental disorders such as autism and microcephaly using cerebral organoids [2, 5]; however, the potential utility of cerebral organoids in regenerative medicine is poorly understood.

In a recent study, an in vivo model of vascularized human brain organoids was developed by transplanting organoids into the superficial cortex of mice [6]. Endogenous blood vessels grew in the human organoids overcame the usual limitation of a lack of vascular circulation. Although widespread axonal extension outside the graft area was observed, region-specific long projections were not reported. Furthermore, the therapeutic potential of organoid transplantation is still not clear. Previously, reported methods produced cerebral organoids containing multiple lumens or neural tubes [2, 7], which makes them difficult to use for transplantation therapy. In addition, massive ventricular zone (VZ) cells lead to a high risk of cell overgrowth, and large organoids might cause more damage to recipients than small organoids if they are transplanted in deep areas of the brain. It is possible that small brain organoids can alleviate these safety concerns and are more amenable to injection into deep brain regions.

The medial prefrontal cortex (mPFC) is a deep region of the cerebral cortex that controls numerous critical brain functions, such as higher cognitive functions, emotions, and goal-directed behaviors [8, 9]. Abnormalities in the mPFC are associated with a variety of human disorders, such as cognitive deficits, drug addiction, autism, and depression [9–12]. Furthermore, the mPFC is involved in many neural circuits that include mPFC-lateral hypothalamus (LH) projections [13]. Thus, transplanting cerebral organoids into the mPFC might result in the reconstruction of neural circuits and offer potential insights into the therapeutic treatment of mPFC-associated disorders.

In this study, we generated small human cerebral organoids based on our previous methods. After transplantation into the mouse mPFC, the cells survived and extended long projections into the LH region after their axons crossed several brain regions. The existence of these long-distance projections was confirmed by injection of a retrograde dye into the LH region. Moreover, we found that the grafted human organoids acquired electrophysiological maturity, formed mutual synaptic connections with host mouse neurons, and potentiated the auditory startle fear response, suggesting that they may functionally integrate into the mouse brain circuits and contribute to physiological functions.

## Materials and methods

### Maintenance of human pluripotent stem cells

hESCs (line H9, WiCell Agreement No. 16-W0060, passage 50–60) and hiPSCs (IMR90-4, WiCell Agreement No. 17-W0063, passage 40–50) were maintained under xeno-free conditions as reported in our previous studies [14, 15]. Half of the medium was exchanged with fresh E8 medium (Thermo Fisher Scientific, Carlsbad, CA, USA) daily, and the cells were passaged every 5–7 days when they reached 80% confluence using ethylenediaminetetraacetic acid (EDTA, Lonza, MA, USA). Before passaging, unhealthy hESC clones were removed manually. In addition, we performed a mycoplasma contamination test every week to ensure the health of all the cell lines (data not shown).

### Small organoid differentiation

To differentiate organoids, hESCs and hiPSCs were detached with dispase (Thermo Fisher Scientific, MA, USA) and then allowed to form embryoid bodies (EBs) the next day according to the methods of our previous report [16]. On the first day, EBs were cultured in half E8 medium and half neural induction medium (NIM; DMEM/F-12, 1% N2, 1% NEAA, Thermo Fisher Scientific, New York, USA). Half of the medium was exchanged with fresh NIM every day for 7 days. The EBs were then attached to six-well plates, and neural tube-like rosettes were observed starting on days 10–11. On day 16, the rosette clones were gently blown off using a 1-ml pipette. The cells were then continuously floated in the same medium until transplantation.

To generate small organoids, organoids were sheared into smaller organoids with a Pasteur pipette (Fisher Scientific, cat. No. 13-678-20D) [17] on day 20 and day 30. Importantly, neural tubes were observed in these sheared organoids over the following 2-3 days. To blow the organoids into even smaller pieces, the end of the Pasteur pipette was passed over a flame to narrow the opening to approximately 0.5 mm in diameter. Then, the narrow part of the shaft was passed over the flame again to create a smooth 15° curve. The organoids were sheared into even smaller spheres of roughly uniform size by passing them through the narrow opening and curve of the pipette.

### Animals and transplantation

All of the animal experiments followed standard protocols provided by the Animal Care and Use Committee at Nanjing Medical University (IACUC1705024-1). Animals were maintained under a 12-h light/dark cycle and provided food and water ad libitum. Six- to eight-week-old severe combined immunodeficient (SCID) mice were anaesthetized with 1.5% isoflurane mixed with oxygen and fixed in a stereotaxic frame. Ten days before transplantation, the organoids were infected with a PGK–GFP lentivirus. To enable precise stimulation of the grafted neurons, donor ChR2–EYFP-expressing hESCs [18] were differentiated into cerebral organoids. Organoids with diameters of approximately 150–250 μm were suspended in 100 μl NIM with B27 (Thermo Fisher Scientific, New York, USA), penicillin–streptomycin solution (HyClone, South Logan, Utah, USA), and a ROCK inhibitor (STEMCELL Technologies, Vancouver, Canada) on the day of transplantation [19]. Approximately, 3–5 small organoids were injected into the mPFC in both hemispheres of each SCID mouse using a glass micropipette angled 15° toward the midline at the following stereotactic coordinates: anterior–posterior = +1.70 mm; lateral to the left = ±1.10 mm; and dorsoventral = −2.7 mm. To compare the effects of different cell formats after transplantation, the same number of organoids were dissociated into single cells (50,000) with TrypLE (Life Technologies, CA, USA) at the same differentiation time (day 40) as small organoids. After surgery, the animals were immediately placed on a prewarmed pad until they became active, at which point they were returned to their home cages.

### Retrograde tracing

Retrograde tracing was performed on SCID mice 3 months after transplantation. After administration of anesthesia, 0.1 μl CTB 555 (Invitrogen, C34776, Bowie, MD, USA) was injected into the bilateral LH at the following stereotactic coordinates: anterior–posterior = −0.82 mm; left to the lateral = ±1.0 mm; and dorsoventral = −5.0 mm. One month later, the animals were perfused with 4% paraformaldehyde (PFA, Sigma, Saint Louis, MO, USA), and histologic analysis was performed.

### Organoids and brain sections

After perfusion, the organoids and brains were fixed with 4% PFA for 0.5–4 h and dehydrated with 20 and 30% sucrose solutions (Sigma, Saint Louis, MO, USA). Organoids were serially sectioned at a thickness of 10 μm. Brains were serially sectioned coronally or sagittally at a thickness of 35 μm and subsequently stored in cryopreservation solution at −20 °C.

### Immunostaining of organoids and brain slices

The organoids and brain slices were washed three times with phosphate-buffered saline (PBS) and blocked for 1 h using 5% donkey serum (Millipore, Saint Louis, MO, USA) and 1% Triton X-100 (Biolink). The slices were incubated in primary antibody diluted with 0.2% Triton X-100 and 5% donkey serum at 4 °C overnight. The next day, the slices were washed three times with PBS and incubated in secondary antibody diluted in 5% donkey serum for 60 min at room temperature in the dark. Finally, the slices were washed and attached to glass slides with Fluoromount-G mounting solution (Southern Biotech). The primary and secondary antibodies are listed in Supplementary Table 2.

### Cell counting and statistical analysis

Images were acquired using a NIKON Eclipse 80i fluorescence microscope and a Zeiss LSM880 microscope with NLO & Airyscan. The fluorescent images were processed and analyzed in ImageJ. For organoid sections, the number of nuclei labeled with Hoechst in each field was counted and recorded as the total cell number. In the transplanted brain sections, the number of grafted cells labeled with human nuclei (hN) was counted and recorded as the total grafted cell number. More than 1000 hN^+^ cells were counted in each section. Student’s *t* test, one-way ANOVA, and two-way ANOVA were used for data analysis. All graphical data are presented as the means ± SEMs and were analyzed by using GraphPad Prism 7.0. For all behavioral experiments, the animals were randomly divided into different groups based on a random number table, and the observers were blinded to the genotypes and treatments during the experiments. Values were considered statistically significantly different at *p* < 0.05 (*), *p* < 0.01 (**), or *p* < 0.001 (***).

### Brain slice preparation and electrophysiological recording

The brains of transplanted mice were removed 3 or 5 months posttransplantation and sliced into 350-µm coronal sections in ice-cold NMDG (N-methyl-d-glucamine)-containing solution consisting of (in mM) 93 NMDG, 93 HCl, 2.5 KCl, 1.2 NaH_2_PO_4_, 30 NaHCO_3_, 20 HEPES, 25 glucose, 5 sodium ascorbate, 2 thiourea, 3 sodium pyruvate, 10 MgSO_4_ and 0.5 CaCl_2_ at pH 7.35 bubbled with 95% O_2_/5% CO_2_ using a Vibratome 2000 (Leica Microsystems). The slices were incubated at 34 °C for 10–15 min in oxygenated NMDG solution, transferred to artificial cerebrospinal fluid (ACSF) containing (in mM) 126 NaCl, 4.9 KCl, 1.2 KH_2_PO_4_, 2.4 MgSO_4_, 2.5 CaCl_2_, 26 NaHCO_3_, and 10 glucose at pH 7.4 at room temperature for approximately 0.5–1 h, and then transferred to a recording chamber containing ACSF bubbled with 95% O_2_/5% CO_2_ at 32 °C. An upright fixed-stage microscope (Olympus) equipped with epifluorescence and infrared-differential interference contrast illumination, a charge-coupled device camera, and two water immersion lenses (10× and 60×) were used to visualize and target GFP^+^ grafted cells. Glass recording electrodes (10–15 MΩ resistance) were filled with an intracellular solution consisting of (in mM) 136 K-gluconate, 6 KCl, 1 EGTA, 2.5 Na2ATP, and 10 HEPES (295 mOsm, adjusted to pH = 7.25 with KOH). Whole-cell patch–clamp recordings were performed using an Olympus microscope (BX51WI), and data were collected and analyzed using an Axopatch1500B amplifier and pCLAMP10 software (Molecular Devices). Alexa Fluor 568 (Invitrogen) was delivered to cells through recording pipettes to evaluate cell morphology. Neurons were clamped at a holding potential of −70 mV, and a series of current steps (10 steps of 10 or 5 pA increments) were injected to elicit APs. Input resistance was calculated from the slope of the current–voltage plot of the change in membrane voltage in response to the series of current injections. The amplitude of the potassium current (*I*_k_) was determined as follows: the total *I*_k_ and fast component of *I*_k_ were measured 250 ms (IK, s) and 7–10 ms (IK, f) after the onset of depolarizing current injection, respectively. Spontaneous postsynaptic currents (sPSCs) were recorded from the grafted human cells at a holding potential of −70 mV in voltage–clamp mode for 3–5 min while they were bathed with 10 μM bicuculline (Abcam, ab146682). For photostimulation of ChR2–EYFP-expressing grafted cells, a blue collimated light-emitting diode with a 470-nm peak wavelength (Mightex BLS) was used. A light intensity of 10–20 mW was applied to ensure effective stimulation. The illumination area was approximately 100 µm^2^ and was centered on the cells selected for stimulation. For all recordings, the leak currents were subtracted using the P/4 procedure. Recordings were low-pass filtered at 2 kHz. Data analysis were performed using Clampfit and GraphPad software. After the recordings were completed, the brain slices were postfixed with 4% PFA in PBS overnight. The data are presented as the means ± SEMs, and significant differences were determined by using Student’s *t* test.

### Behavioral tests

#### Open field test

Mice were placed in a plastic box (50 cm long × 50 cm wide × 50 cm high) and allowed to freely explore the arena under standard overhead lighting conditions [20]. The behavioral performance of the mice was recorded for 6 min with an infrared digital camera (Clever Sys. Inc.). Quantitative analysis (TopScan Version 3.0) of the total distance traveled, time spent in the center, and the number of crossings through an arbitrarily defined center zone (25 cm × 25 cm) was performed.

#### Fear conditioning

The SCID mice were trained and tested on a Video Fear Conditioning System (superfcs 2.0) with a floor consisting of a metal rod connected to a shock generator (XR-XC404). Each chamber was individually enclosed in a sound-attenuating cubicle for the behavioral test [21]. During the training, the SCID mice were fear conditioned to six 30-s, 10-kHz, 75-dB tones (conditioned stimuli), each terminating with a 2-s, 0.7-mA foot shock (unconditioned stimulus). The interval between each unconditioned stimulus was 30 s. The chamber was cleaned before each training session with 75% ethanol. At the conclusion of the conditioning session, the mice were returned to their cages.

## Results

### Generation of small cerebral organoids

**Fig. 1:**
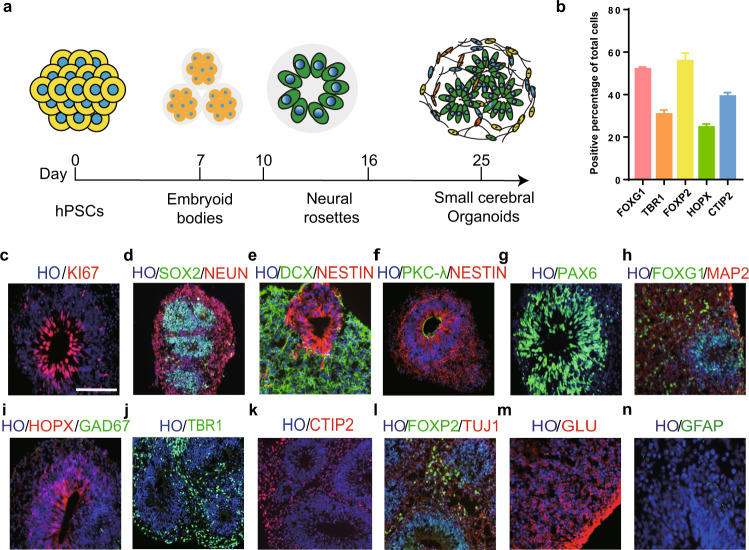
The differentiation of small human cerebral organoids in vitro. **a** Schematic of the brief timeline of the generation of small human cerebral organoids from human pluripotent stem cells. **b** Quantification of cortical cell populations in vitro at day 30 postdifferentiation (*n* = 30 organoids). **c**, **g** Representative immunostaining of human cerebral organoids for Ki67 (a proliferation marker) and PAX6 (a dorsal telencephalic progenitor marker) at 30 days postdifferentiation. **d** Double immunostaining for the ventricular zone (VZ) marker SOX2 and the mature neuronal marker NeuN. NeuN^+^ cells were found in the peripheral region of cerebral organoids. **e**, **f** Immunostaining for DCX (a newborn neuron marker), PKC-λ (an adherent junction marker), and Nestin (a neural stem cell marker). Few DCX^+^ and Nestin^+^ cells were found. **h**–**l** Sample images of immunostaining for the early stage forebrain marker FOXG1, the oRGC marker HOPX, the GABAergic interneuron marker GAD67, the cortical-layer neuron markers TBR1, CTIP2, and FOXP2, and neuronal markers TUJ1 and MAP2 in cerebral organoids. **m** Human cerebral organoids derived from human pluripotent stem cells expressed glutamate (a glutamatergic neuronal marker) in our culture system. **n** Glial fibrillary acidic protein (GFAP, human astrocytic marker) was expressed at a low level in cerebral organoids. Scale bars: 100 μm.

Breakthroughs have been made in differentiating brain/cerebral organoids based on the self-organizing capacity of hESC/hiPSC-derived neural epithelial cells [4, 22]. Recently reported methods have yielded large organoids containing multiple neural tubes, but challenges remain due to the limited nutrient absorption and reduced maturation rates of the cells deep inside these large organoids. To generate small organoids, we differentiated cells by using our previously developed methods [16, 17, 19] (Fig. 1a). To control the size of the organoids, which ranged from 150 to 250 µm, we sheared the organoids at day 20 and day 30 with a glass pipette (Supplementary Fig. 1c, d). On day 40, the percentage of organoids with a single neural tube in the culture system was approximately 40–50%, and the remaining organoids were small and had only 3–5 neural tubes (Supplementary Fig. 1a, b). Because the small organoids were of a controlled size, a spinning bioreactor or spinner was not required to enhance nutrient absorption. Furthermore, in contrast to large organoids, small organoids did not exhibit cavities when maintained in culture (currently up to 100 days, Supplementary Fig. 1e).

In the early stages (25–30 days in culture), the small organoids expressed KI67 and SOX2 in the VZ and subventricular zone (SVZ) (Fig. 1c, d). The neural stem cell marker NESTIN was uniquely expressed in the VZ, while the newborn neural marker DCX was expressed in the outer layers (Fig. 1e). The apical marker PFC-λ was expressed in the inside loop, which was surrounded by NESTIN^+^ cells (Fig. 1f). The cortical progenitor marker PAX6 (Fig. 1g) and the telencephalic marker FOXG1 were strongly expressed in the neural tube (in approximately 50% of all cells, Fig. 1b, h). In the late stages (40–50 days in culture), the outer SVZ marker HOPX was expressed in the lumen (in approximately 25% of all cells, Fig. 1b, i). The superficial cortical marker TBR1 was expressed in the outer layers of the lumen (in approximately 30% of all cells, Fig. 1b, j). The layer V/VI markers CTIP2 and FOXP2 were also expressed in the upper layers of the organoids (in approximately 40 and 60% of all cells, respectively) (Fig. 1b, k, l). The vast majority of neurons in the organoids were positive for glutamate (Fig. 1m) but not GAD67, a GABAergic interneuron marker, suggesting that the organoids displayed characteristics of dorsal telencephalic neurons. In addition, a small proportion of cells (less than 1%) in the organoids were GFAP^+^ (Fig. 1n). Furthermore, the small organoid differentiation protocol was repeated in IMR90-4 iPSCs (Supplementary Fig. 2). Taken together, the immunostaining results indicate that we successfully generated small human cerebral organoids with a small number of neural tubes (fewer than five) via our previously described protocols [16, 17].

### Grafted human organoids in the mPFC exhibited cortical differentiation

**Fig. 2:**
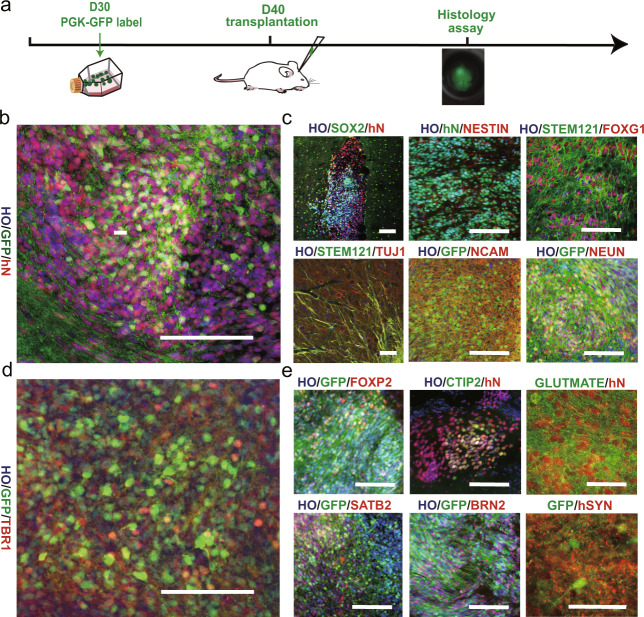
The growth of small human cerebral organoids after transplantation in vivo. **a** After 30 days postdifferentiation, human cerebral organoids were labeled with PGK-GFP. Ten days later, GFP^+^ cerebral organoids were grafted into the brains of immunodeficient mice and harvested at the indicated MPT (month posttransplantation) for histological analysis (1 MPT: *n* = 5 brains; 3 MPT: *n* = 8 brains). **b** Immunofluorescence staining for GFP and human nuclei (hN) demonstrating that the implant survived well and was distributed throughout the prefrontal cortex by 1 MPT. **c** One month later, the expression of SOX2, FOXG1, STEM121 (a human neurite marker), hN, NCAM (a human neural cell adhesion molecule), NEUN, and TUJ1 were detected in the mouse brain. **d**, **e** More than 2 months later, the expression of TBR1, FOXP2, SATB2, BRN2, and glutamate (GLU) indicated that cortical excitatory features were conserved in vivo. In addition, a representative image of immunostaining for GFP and human-specific synaptophysin (hSyn) after 3 MPT is shown. Scale bars: 100 μm.

To examine the therapeutic potential of human small cerebral organoids, we transplanted them into the mouse mPFC, a deep region of the cortex that is critical for controlling higher behaviors [23]. The organoids were labeled with a PGK-GFP lentivirus around day 30 and were injected into the mPFC on day 40 with a pulled glass pipette (Fig. 2a).

At 1–3 months, we analyzed the differentiation of the grafted organoids in the mouse brain. Human cells were identified in the host brain based on GFP, hN, and STEM121 expression. All GFP^+^ cells were also hN^+^ (Fig. 2b), demonstrating that all of the grafted GFP^+^ cells were human cells. At 1 month posttransplantation, no NANOG^+^ cells were observed among the hN^+^ cells (Supplementary Fig. 3a), suggesting that there were no pluripotent stem cells among the grafted cells. KI67^+^ cells were rarely observed (Supplementary Fig. 3b), indicating that nearly all grafted cells had exited the cell cycle. Approximately, 30% of the hN^+^ cells were also SOX2^+^ cells (Fig. 2c). At 3 months posttransplantation, less than 1% of the cells were NESTIN^+^ (Fig. 2c), indicating that most of the grafted human cells had become postmitotic. Furthermore, more than 80% of the human cells were positive for FOXG1, TUJ1, and NCAM (Fig. 2c), demonstrating that the majority of the grafted human cells were forebrain neurons, and approximately 50% of the GFP^+^ cells also expressed NEUN (Fig. 2c), suggesting that the transplanted human cells gradually matured over 3 months.

We next determined the identity of the grafted cerebral organoids by analyzing cortical layer markers. At 3 months posttransplantation, the grafted cells expressed cortical biomarkers such as TBR1, FOXP2, CTIP2, SATB2, and BRN2 (Fig. 2d, e), demonstrating that the organoids had characteristics of cortical neurons. Furthermore, glutamate, VGLUT1, and human synaptophysin staining showed that the grafted human organoids were glutamatergic and tended to be mature (Fig. 2e, Supplementary Fig. 3c). In addition, 5% of the grafted cells were positive for GFAP (Supplementary Fig. 3d), showing that some of the cells in the grafted organoids had differentiated into astroglial cells. Immunostaining for PDGFR-a and MBP showed that a small proportion of the grafted cells had differentiated into oligodendrocytes, suggesting that the cells had started to undergo myelination (Supplementary Fig. 3e, f). Similar results were observed by engrafting IMR90-4 iPSC-derived small organoids, confirming our findings (Supplementary Fig. 4).

Taken together, these results show that the transplanted small human organoids survived, became postmitotic after one month and differentiated into cortical neurons in multiple layers in vivo.

### Engrafted organoids differentiated into functionally mature neurons in the host brain

**Fig. 3:**
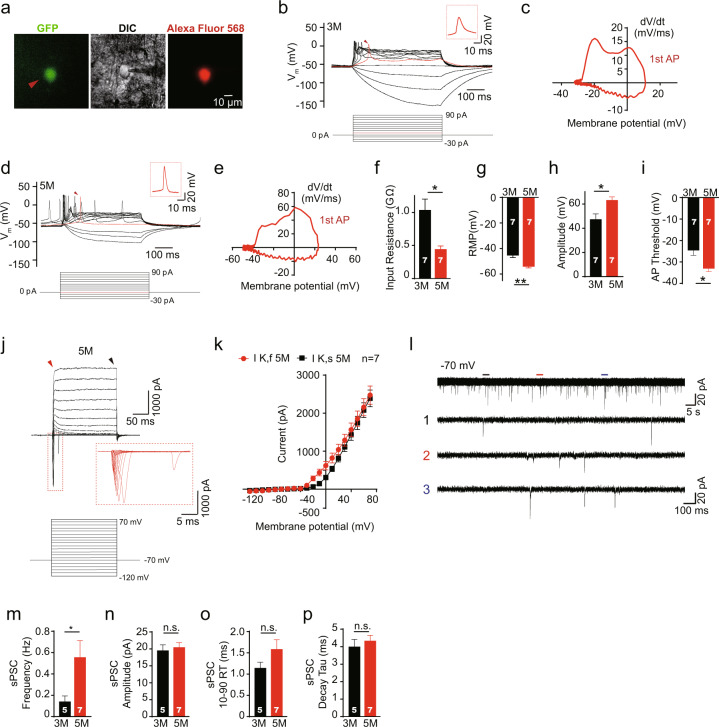
Grafted neurons display electrophysiological characteristics of mature neurons. **a** Representative images of a recorded EGFP-expressing neuron at 5 months after transplantation. The EGFP^+^ cell (left, red arrowhead) was recorded in the whole-cell configuration guided by DIC microscopy (*middle*) and then filled with Alexa Fluor 568 dye (right). **b** Representative traces showing the changes in the membrane voltage of an EGFP-expressing neuron in response to a series of step current injections at 3 months after transplantation. Note that the highlighted traces (red, upper traces) show the first action potential generated in response to a 10-pA injection (red, lower traces). **c** A phase plot of the *I*–*V* relationship of the EGFP-expressing neuron in (**b**). **d**, **e** Same as (**b**) and (**c**), but at 5 months posttransplantation. Note that the first AP was generated by a 0-pA injection (red arrowhead). The boxed insets in (**b**) and (**d**) show the expansion of the first AP (arrowhead) on an enlarged scale. **f**, **i** Summary of the input resistance (**f**), resting membrane potential (RMP) (**g**), action potential peak amplitude (**h**), and threshold (**i**) of EGFP-expressing neurons at 3 months and 5 months after transplantation. *p < 0.05; **p < 0.005, Student’s *t* test. **j** Representative traces of Na^+^ and K^+^ currents evoked in EGFP-expressing neurons from −120 to 70 mV in 10-mV steps at 5 months posttransplantation. The boxed inset shows the expansion of Na^+^ currents on an enlarged scale. The red arrowhead indicates the fast component of the K^+^ currents (IK, f), and the black arrowhead indicates the slow component of K^+^ currents (IK, s). **k** The *I*–*V* plot of averaged outward currents corresponding to (**j**). **l** Sample traces of spontaneous postsynaptic currents (sPSCs) at a holding potential of −70 mV from an EGFP-expressing neuron at 5 months after transplantation. The bottom three traces are expanded from the top traces drawn with thick lines. **m**–**p** Summary of the frequency (**n**), amplitude (**o**), rise time (**p**), and decay time (**q**) of sPSCs recorded at a holding potential of −70 mV from neurons at 3 months or at 5 months after transplantation. *p < 0.05, Student’s *t* test. The data are presented as the mean ± SEM. *N* indicates the number of neurons recorded.

To examine the electrophysiological properties of neurons from the engrafted organoids, we next performed whole-cell patch–clamp recordings of enhanced green fluorescent protein (EGFP)-expressing neurons in the mouse cortex at 3 and 5 months posttransplantation (Fig. 3a). We found that the EGFP-expressing neurons reliably generated action potentials (APs) in response to membrane depolarization at 3 months posttransplantation (Fig. 3b–e). Compared with those observed at 3 months posttransplantation, engrafted human cells observed at 5 months posttransplantation showed a more negative resting membrane potential and AP threshold, a decrease in input resistance and an increase in AP amplitude (Fig. 3b–i), suggesting that they progressively matured as time passed after transplantation. Large inward sodium currents were evoked in response to membrane depolarization, and typical *I*–*V* curves for potassium channels were also observed in the engrafted EGFP-expressing neurons, indicating that they acquired electrophysiological maturity (Fig. 3j, k).

To further investigate whether the EGFP-expressing engrafted neurons had become integrated into pre-existing circuits in the host mouse brain, we recorded sPSCs from the EGFP-expressing neurons (Fig. 3l). sPSCs were observed in EGFP-expressing neurons at 3 months posttransplantation. Importantly, although the amplitude, rise time, and decay time of the sPSCs were not changed before transplantation and at 5 months posttransplantation (Fig. 3n–p), the frequency was significantly increased at 5 months posttransplantation (Fig. 3m). Together, these data suggest that neurons derived from grafted human organoids rapidly underwent functional maturation based on electrophysiological properties and synaptic connections in the mouse brain.

### Grafted human cerebral organoids formed bidirectional synaptic connections with the host mouse brain

**Fig. 4:**
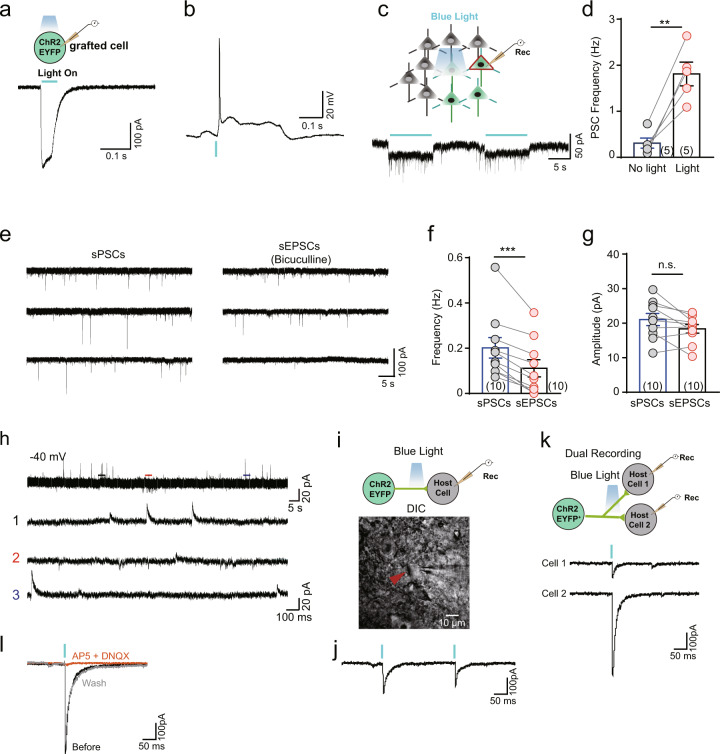
Grafted human cerebral organoids expressing ChR2–EYFP are functionally integrated into synaptic circuits in the host mouse brain. **a** Schematic of a whole-cell recording of a grafted cell at 5 months after transplantation (top) and ChR2-mediated currents elicited in the grafted cells by blue light illumination (bottom). **b** An action potential triggered in a grafted cell by activation of ChR2. **c**, **d** Photostimulation of grafted cells expressing ChR2 increased the frequency of postsynaptic currents (PSCs). Schematic of the experimental paradigm (**c**, top), a representative trace from a grafted cell, and quantification of sPSCs with light off and light on (**c**, bottom). **p < 0.01, Student’s *t* test. **e** Sample traces of spontaneous PSCs (left) and spontaneous excitatory postsynaptic currents (sEPSCs, right) recorded in a grafted cell (left) at 5 months after transplantation at a holding potential of −70 mV. sEPSCs were recorded in buffer containing bicuculline (10 µM, right). **f**, **g** Quantification of the frequency (**f**) and amplitude (**g**) of sPSCs and sEPSCs from ChR2-expressing neurons at 5 months after transplantation. ***p < 0.001, Student’s *t* test. **h** Sample traces of spontaneous postsynaptic currents (sPSCs) at a holding potential of −40 mV from an EYFP-expressing neuron at 5 months after transplantation. The bottom three traces are expansions of the areas of the top traces drawn with thick lines. **i** Schematic of the experimental paradigm and a DIC image of a recorded host cell in the cortex. Photostimulation activated ChR2-expressing neurons that generated excitatory postsynaptic currents in a recorded EYFP-negative host cell. **j** Sample trace of two PSCs elicited in a host cell by paired pulses of light stimulation at a holding potential of −70 mV. **k** Dual recordings showing PSCs simultaneously evoked in a pair of host cells in response to photostimulation. **l** Sample trace of light-evoked PSCs recorded in a host cell before (black) and after (orange) application and after washout (gray) of AP5 (50 μM) and DNQX (10 μM), which block ionotropic glutamatergic receptors. The data are presented as the mean ± SEM. *N* indicates the number of neurons recorded.

We next asked whether synaptic connections formed among the grafted human organoids or between the grafted human organoids and mouse neurons. Glutamatergic organoids were similarly generated from a previously reported hESC line expressing channelrhodopsin 2 (ChR2) fused with EYFP [18]. Five to 6 months after these organoids were transplanted into the mPFC of NOD-SCID mice, blue light illumination reliably elicited a very large ChR2-mediated inward current (Fig. 4a) or an AP (Fig. 4b) in the grafted EYFP^+^ cells. Then, a spot of light was applied to stimulate ChR2-expressing axons surrounding the dendrites and soma of the recorded grafted neurons. In addition to the small inward currents elicited in the recorded neuron (Fig. 4c), we found an increase in the frequency of postsynaptic currents in the recorded grafted neurons following light stimulation (Fig. 4c, d), suggesting that synaptic connections were established among the cells of the grafted human cerebral organoid.

Grafted glutamatergic human organoids received glutamatergic inputs from both grafted human neurons and host mouse neurons, but GABAergic organoids received inputs exclusively from mouse cells. We found that blockage of the type A gamma-aminobutyric acid receptor (GABA_A_R) with bicuculline reduced the frequency of sPSCs by ~50% but had no effect on the amplitude (Fig. 4e–g). Moreover, a number of sPSCs recorded using a recording pipette solution containing Cl^-^, which has a reversal potential of approximately −65 mV, became outward currents at a holding potential of −40 mV (Fig. 4h). These results indicate that grafted human organoids appeared to receive GABAergic projections from mouse neurons.

To further examine whether the grafted human organoids could form mutually synaptic connections with mouse neurons, we took advantage of optogenetic approaches to selectively stimulate the ChR2–EYFP^+^ axons of grafted human organoids that surrounded a recorded mouse cortical neuron (Fig. 4i). We found that short repeated pulses of spots of blue light illumination (10–50 ms, 20 CW) evoked robust inward postsynaptic currents in approximately 30% of mouse cortical neurons (3 of 10 cells) at a holding potential of −70 mV (Fig. 4j, k). These currents were completely blocked by bath application of AP5 and DNQX, which are NMDA and AMPA receptor antagonists, respectively, and recovered after washing out the substances (Fig. 4l), indicating that they were glutamatergic postsynaptic currents. Taken together, these results demonstrate that our grafted human cerebral organoids formed bidirectional synaptic connections with host mouse cortical neurons.

### Engrafted organoids extended long-distance projections into basal brain regions

**Fig. 5:**
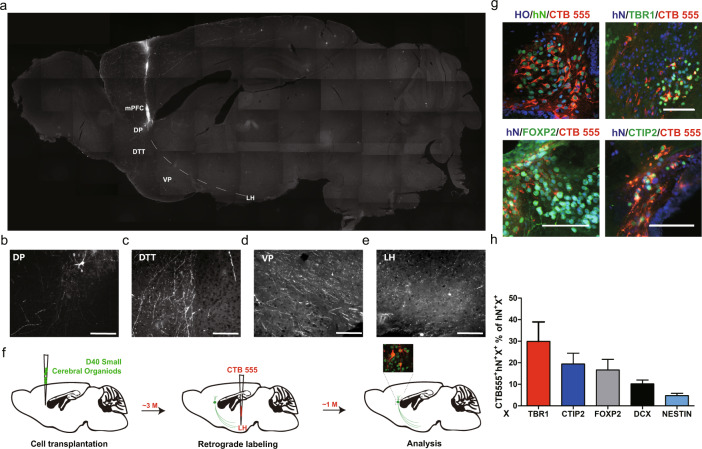
The transplanted cerebral organoids successfully extended long-distance subcortical axonal projections to basal brain regions. **a** Slide scanner images of GFP-stained sagittal brain sections obtained from a grafted mouse brain at 3 MPT showing long projections (*n* = 3 mice). **b**–**e** High-magnification views of the sampled regions are shown in (**a**). DP dorsal peduncular cortex, DTT dorsal tenia tecta, VP ventral pallidum, LH lateral hypothalamus. **f** Illustration of the timeline of cell transplantation, retrograde labeling, and analysis. **g** The retrograde tracer CTB 555 reached the grafted cells and was coexpressed with hN (upper left). A representative image of immunostaining for the cortical projection neuron markers TBR1 (upper right), FOXP2 (bottom left), and CTIP2 (bottom right), each of which was coexpressed with CTB555. Scale bar: 100 µm. **h** Quantification of CTIP2^+^, TBR1^+^, FOXP2^+^, DCX^+^, NESTIN^+^, and SOX2^+^ cell populations in the CTB 555^+^/hN^+^ graft area (*n* = 3 mice, mean ± SEM). Scale bars: 100 µm.

Region-specific projections from human-derived neural cells/tissues could potentially reconstruct brain circuits and permit functional repair. To determine whether engrafted small cerebral organoids produced neurite outgrowths, we analyzed brain sections in regions other than the injection area. Notably, at 1 month posttransplantation, extensive STEM121^+^ fibers were found in coronal sections of the LH, a basal brain structure (Supplementary Fig. 3h). We next investigated sagittal brain sections. Bundles of GFP^+^ neurites that projected from the mPFC toward the LH, crossing multiple brain regions, were observed within the first month after transplantation (Fig. 5a). The lengths of the projections were over 4.5 mm at 1 month posttransplantation, and immunostaining for GFP showed a high density of human neurites in each section along the projection pathway in the LH (Fig. 5b–e). Notably, human-specific synaptophysin (hSYN) was observed in the LH and was coexpressed with multiple other neuronal markers, such as MCH, VGLUT1, and GABA, indicating that the long-projection human neurites may have formed functional synapses with LH neurons (Supplementary Fig. 3i–k). Similar human projections were also found in the LH in the group transplanted with IMR90-4-derived organoids (Supplementary Fig. 4b). Thus, the data revealed that a large number of long projections that extended from engrafted small cerebral organoids was found in the LH at 1 month posttransplantation.

To further confirm the existence of long projections from neurons in the mPFC, we injected cholera toxin subunit B (CTB 555), a retrograde axonal tracer bound to axonal bundles [24–26], into the LH at 3 months posttransplantation (Fig. 5f). One month later, the presence of the retrograde tracer in coronal sections was examined. Strikingly, we found colabeling of hN and CTB 555 in both the mPFC and LH, suggesting that human neurites in the LH stemmed from transplanted organoids in the mPFC (Fig. 5g).

Different populations of cortical neurons in different brain regions extend unique projections to distal brain regions [27, 28]. To identify the subclass of cortical neurons that sent neurite projections into the LH, we performed immunostaining of brain sections from the injection site. We found that 30% of the TBR1^+^/hN^+^ cells were CTB 555^+^, while 17% of the retrograde labeled human cells were FOXP2^+^, and 20% were CTIP2^+^ (Fig. 5g and Supplementary Fig. 3g). The remaining projection cells that were not TBR1^+^, CTIP2^+^, or FOXP2^+^ were DCX^+^ and NESTIN^+^. FOXP2 and CTIP2 are cortical markers of layer V neurons, while TBR1 is a marker of layer VI neurons, indicating that the majority of the projections in the LH were extended by potential neurons in the grafted organoids from the mPFC.

**Fig. 6:**
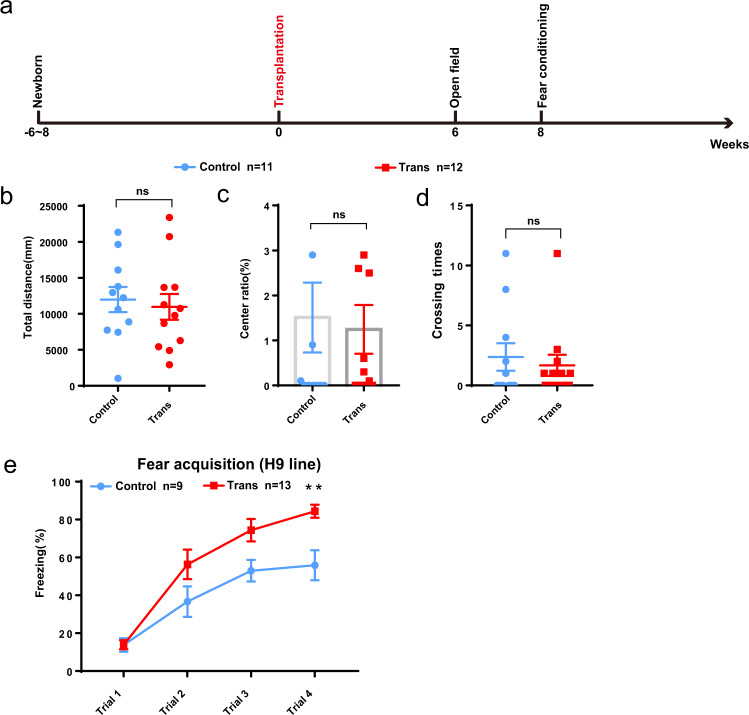
Human cerebral organoid transplantation potentiates the startle fear response. **a** Schematic presentation of the time course of transplantation and behavioral analysis. **b**–**d** The open field test indicated no difference in the time spent in the center, number of center crossings, or total distance traveled between organoid-injected mice and control mice injected with cerebrospinal fluid (control: *n* = 11 mice; transplantation: *n* = 12 mice). **e** The transplantation of cerebral organoids promoted the freezing response of the mice to a tone (control: *n* = 9 mice; transplantation: *n* = 13 mice). The data are shown as the mean ± SEM.

To examine whether the engrafted organoids contributed to host behavior, we performed the open field and fear conditioning tests at 6 weeks and 8 weeks posttransplantation, respectively (Fig. 6a). NOD-SCID mice that received injected with cerebrospinal fluid (CSF) were used as controls. Analysis of the open field test data showed that the total distance traveled, the proportion of time spent in the center, and the number of crossings by the organoid-engrafted animals were similar to those by the control animals (Fig. 6b–d), suggesting that small organoid transplantation did not impair the normal physiological function of the mice.

Emotional and episodic memory is partially regulated by glutamatergic neurons in the mPFC [9, 23, 24, 29]; thus, we asked whether fear learning and memory were affected after engrafting human glutamatergic neurons into this region. Notably, the percentage of freezing in the fear conditioning test was higher in the grafted group than in the control group (Fig. 6e), and this finding was confirmed in another independent transplantation study involving IMR90-4 iPSC-derived small organoids (Supplementary Fig. 4). We transplanted another group of animals with dissociated neural cells and compared them to the animals transplanted with small cerebral organoids. The fear acquisition of the dissociated neural cell group was not different from that of the control group (Supplementary Fig. 5a), as there were fewer surviving cells in the dissociated neural cell group than the small organoid group (Supplementary Fig. 5b, c).

Together, our behavioral results show that while motor ability was not altered in the grafted mice, the startle fear response was potentiated by human organoid transplantation.

## Discussion

Cerebral organoids are powerful tools for studying human brain development and neurological diseases. However, the potential for the reconstruction of neural circuits remains to be investigated. In this study, we developed an improved approach for obtaining small cerebral organoids containing fewer than five neural tubes. After transplantation, the human organoids extended long projections into distal brain regions within one month and acquired cerebral cell characteristics within 3 months and electrophysiological maturity within 5 months. Interestingly, neurons rapidly differentiated from the transplanted human organoids and became functionally integrated into mouse neural circuits via the formation of mutual synaptic connections with mouse neurons; furthermore, the host mice that received human organoid transplants showed an enhanced startle fear response to an auditory conditioned stimulus, which may have been due to potentiation of fear memory acquisition [30]. Although previous studies showed that oscillatory electrical waves are periodically and regularly generated in in vitro cultured human organoids [31], this study demonstrated that transplanted human organoids can form bidirectional synaptic connections with host neurons and play an important role in higher-order brain functions.

Unlike in previous studies, we injected a limited number of small organoids into a basal brain region and observed point-to-point projections through and into deep brain regions. Indeed, in contrast to the massive grafts that are normally observed after transplantation [32, 33], we observed grafts of a controlled size and only a few proliferative cells in the host brain, indicating that our small cerebral organoids are comparatively safe for future therapeutic applications. In addition to the projections that extended into the cortex from grafted cells along the needle track, robust projections were observed in the LH at one month posttransplantation; this time point is earlier than what was reported in previous studies [6] and correlates well with the alteration in mouse behavior. Since human fibers were observed in other brain areas, such as the parietal lobe, we cannot exclude the possibility that the projections throughout the cortex also contributed to improved fear acquisition. Although the open field test showed that transplantation did not induce behavioral impairments in the mice, the results could not rule out other effects of transplantation on brain function. Furthermore, a small population of STEM121^+^ cells coexpressed MBP, suggesting that human myelination may have occurred in the brains of mice that underwent transplantation.

Projections from human grafts have been reported by several groups [32, 34, 35], but long subcortical glutamatergic projections have been rarely observed. In the current study, the potential neurons, including TBR1^+^, FOXP2^+^, and CTIP2^+^ neurons in the grafted cerebral organoids, extended long projections from the mPFC into basal brain regions that followed intrinsic projection pathways, suggesting that the correct neural subtype is crucial for specific transplantation therapies. Indeed, our previous study demonstrated that human GABAergic neurite projections extend from the medium septum to the hippocampus [36]. Therefore, grafted neurons can undergo cellular–anatomic integration, hinting that the induction of functional recovery by transplantation requires the correct neural subtype. Inappropriate neural subtypes might form heterotopic projections and have unpredictable effects [37]. The existence of projections in the LH were confirmed by a retrograde tracing experiment; however, the functional connections made with the LH were hard to detect, as long-range axons derived from grafted human organoids rarely remained intact during the preparation of mouse brain slices.

Together, our findings demonstrated that small human cerebral organoids survived and rapidly underwent maturation based on electrophysiological membrane properties and the formation of synapses after transplantation and that the grafted organoids extended long projections through different brain regions. Our research might open novel avenues for future transplantation studies based on human brain organoids.

## Supplementary information

Supplementary Materials for Human cerebral organoids establish subcortical projections in the mouse brain after transplantation

Supplementary Fig. 1

Supplementary Fig. 2

Supplementary Fig. 3

Supplementary Fig. 4

Supplementary Fig. 5

Supplementary figure legends
