# Supplementary material for: Human cerebral organoids establish subcortical projections in the mouse brain after transplantation
Source: Mol Psychiatry. 2020 Oct 13;26(7):2964–76. doi: 10.1038/s41380-020-00910-4 (PMC8505255; doi:10.1038/s41380-020-00910-4)
Supplement: Supplementary file 1 — Supplementary Materials for Human cerebral organoids establish subcortical projections in the mouse brain after transplantation [file 41380_2020_910_MOESM1_ESM.pdf]

Supplementary Materials for

Human cerebral organoids establish subcortical projections in the mouse brain after transplantation

Xin Dong[1,2#], Shi-Bo Xu[1#], Xin Chen[3#], Meng-Dan Tao[1], Xiao-Yan Tang[1], Kai-Heng Fang[1], Min Xu[1], Yufeng Pan[4], Yuejun Chen[5], Shuijin He[3*], and Yan Liu[1,6*]

*Correspondence: yanliu@njmu.edu.cn or heshj@shanghaitech.edu.cn

# These authors contributed equally to this work

This PDF file includes:

Supplementary Fig. 1-5

Supplementary Table 1-3

References

Supplementary Table 1

Overview of different transplantation trials based on stem cell therapy.

hPSC-derived material	Depth of transplantation	Projection area	Distance of the projections	Electrophysiological activity	Functional integration	Behavioral improvement	Ref
Cerebral organoids	Retrosplenial cortex (DV: -0.2~1 mm)	Ventrally and rostrally innervated more in the ipsilateral hemisphere	Almost 4 mm (at 90 dpi)	Yes, multielectrode array results suggested functional maturation at the late stage	Yes, optogenetic stimulation revealed functional output connectivity from the organoid graft to the host brain.	No significant change	1
Single cortical neurons	Somatosensory cortex (DV: -0.5 mm)	Ipsilateral motor cortex, striatum, thalamus, contralateral SCx1 and the corpus callosum	In the centimeter range (at 5 MPT)	Yes, grafted neurons were excitable, fired action potentials and received both excitatory and inhibitory inputs.	Yes, electron microscopy and ex vivo whole-cell recording suggested structural integration and functional network connectivity.	None	2
Dissociated neural progenitor cells and organoids	1 mm below the dura	None	None	None	None	None	3
Small cerebral organoids vs dissociated neural cells	mPFC (DV: -2.6 mm)	From mPFC to LH and to more ventral and caudal brain areas	More than 4.5 mm (at 1 month)	Yes, engrafted organoids differentiated into functionally mature neurons	Yes, whole-cell recordings with channelrhodopsin 2 stimulation revealed an integrated network and functional postsynaptic integration.	Yes, fear memory acquisition was increased	This study

Supplementary Table 2: Antibodies used in this study.

Antibody	Isotype	Dilution	Source (cat.no)
BRN2	Mouse IgG	1:500	Santa Cruz (sc-393324)
CTIP2	Rat IgG	1:400	Abcam (ab1845）
DCX	Rabbit IgG	1:300	Cell Signal Technology (4604)
FOXP2	Rabbit IgG	1:1000	Abcam (AB16046)
FOXG1	Rabbit IgG	1:1000	Abcam (AB18259)
GFAP	Rabbit IgG	1:2000	Dako (Z0334)
GFP	Rabbit IgG	1:1000	Chemicon (AB3080)
Glutamate	Rabbit IgG	1:5000	Sigma-Aldrich (G6642)
Human Nuclei	Mouse IgG	1:1000	Chemicon (MAB1281)
HOPX	Rabbit IgG	1:500	Sigma-Aldrich (HPA030180)
Human Synaptophysin	Human IgG	1:500	eBioscience (147-6525-80)
Ki67	Rabbit IgG	1:500	ZYMED (180191Z)
MAP2	Mouse IgG	1:1000	Sigma-Aldrich (M1406)
NANOG	Goat IgG	1:1000	R&D (AF1997)
NeuN	Mouse IgG	1:2000	Arigo (ARG53383)
NESTIN	Goat IgG	1:1000	Santa Cruz (sc-21247)
NCAM	Mouse IgG	1:1000	Santa Cruz (SC-106)
PAX6	Rabbit IgG	1:1000	Covance (PRB-278P)
PDGFra	Rabbit IgG	1:500	Cell Signal Technology (3174s)
PKC-λ	Mouse IgG	1:1000	BD (610207)

VGLUT1	Mouse IgG	1:1000	Sysy (135311)
SATB2	Mouse IgG	1:1000	Abcam (AB51502)
Sox2	Goat IgG	1:300	R&D (AF2018)
TBR1	Rabbit IgG	1:1000	Abcam (AB31940)
STEM121	Mouse IgG	1:1000	Stem Cell (AB-121-U-050)
β-III Tubulin	Mouse IgG	1:2000	Sigma-Aldrich (T8660)
β-III Tubulin	Rabbit IgG	1:2000	Covance (PRB-435P)

**Antibody**	**Dilution**	**Source (cat.no)**
488 Donkey Anti-Goat	1:2000	Thermo Fisher Scientific (A11055)
488 Donkey Anti-Mouse	1:2000	Thermo Fisher Scientific (A21202)
488 Donkey Anti-Rabbit	1:2000	Thermo Fisher Scientific (A21206)
546 Donkey Anti-Goat	1:2000	Thermo Fisher Scientific (A11056)
546 Donkey Anti-Mouse	1:2000	Thermo Fisher Scientific (A10036)
546 Donkey Anti-Rabbit	1:2000	Thermo Fisher Scientific (A10040)

Supplementary Table 3.

Summary table showing the number of immunodeficient mice used for immunostaining, behavioral, electrophysiological and retrograde tracing experiments.

Figure number	Group	Transplantation time	Brain sections	Number of mice
Fig. 2b and 2c, Supplementary Fig. 3a-f	N/A	> one month	Sagittal	n=5
Fig. 2d and 2e	N/A	2~3 months	Sagittal	n=8
Fig. 3b	N/A	3 months	Coronal	n=3
Fig. 3d	N/A	~5 months	Coronal	n=5
Fig. 4	N/A	~5 months	Coronal	n=3
Fig. 5a-e	N/A	2~4 months	Sagittal	n=8
Fig. 5g	N/A	>4 months	Sagittal	n=6
Fig. 6b-d	Control	>1.5 months	N/A	n=11
	Trans	>1.5 months	N/A	n=12
Fig. 6e	Control	>1.5 months	N/A	n=9
	Trans	>1.5 months	N/A	n=13
Supplementary Fig. 3h-k	N/A	1~2 months	Coronal	n=3
Supplementary Fig. 4	N/A	2~3 months	Sagittal	n=5
Supplementary Fig. 5a	Control	8 weeks	N/A	n=9
	DISS	8 weeks	N/A	n=9
	SMAL	8 weeks	N/A	n=14
Supplementary Fig. 5 b-c	N/A	1~2 months	Sagittal	n=8

References

1. Mansour AA, Goncalves JT, Bloyd CW, Li H, Fernandes S, Quang D *et al.* An in vivo model of functional and vascularized human brain organoids. *Nat Biotechnol* 2018; **36**(5)**:** 432-441.

2. Real R, Peter M, Trabalza A, Khan S, Smith MA, Dopp J *et al.* In vivo modeling of human neuron dynamics and Down syndrome. *Science* 2018; **362**(6416).

3. Daviaud N, Friedel RH, Zou H. Vascularization and Engraftment of Transplanted Human Cerebral Organoids in Mouse Cortex. *eNeuro* 2018; **5**(6).
