# Supplementary figures and images for: Human cerebral organoids establish subcortical projections in the mouse brain after transplantation
Source: Mol Psychiatry. 2020 Oct 13;26(7):2964–76. doi: 10.1038/s41380-020-00910-4 (PMC8505255; doi:10.1038/s41380-020-00910-4)

Supplementary Fig. 1

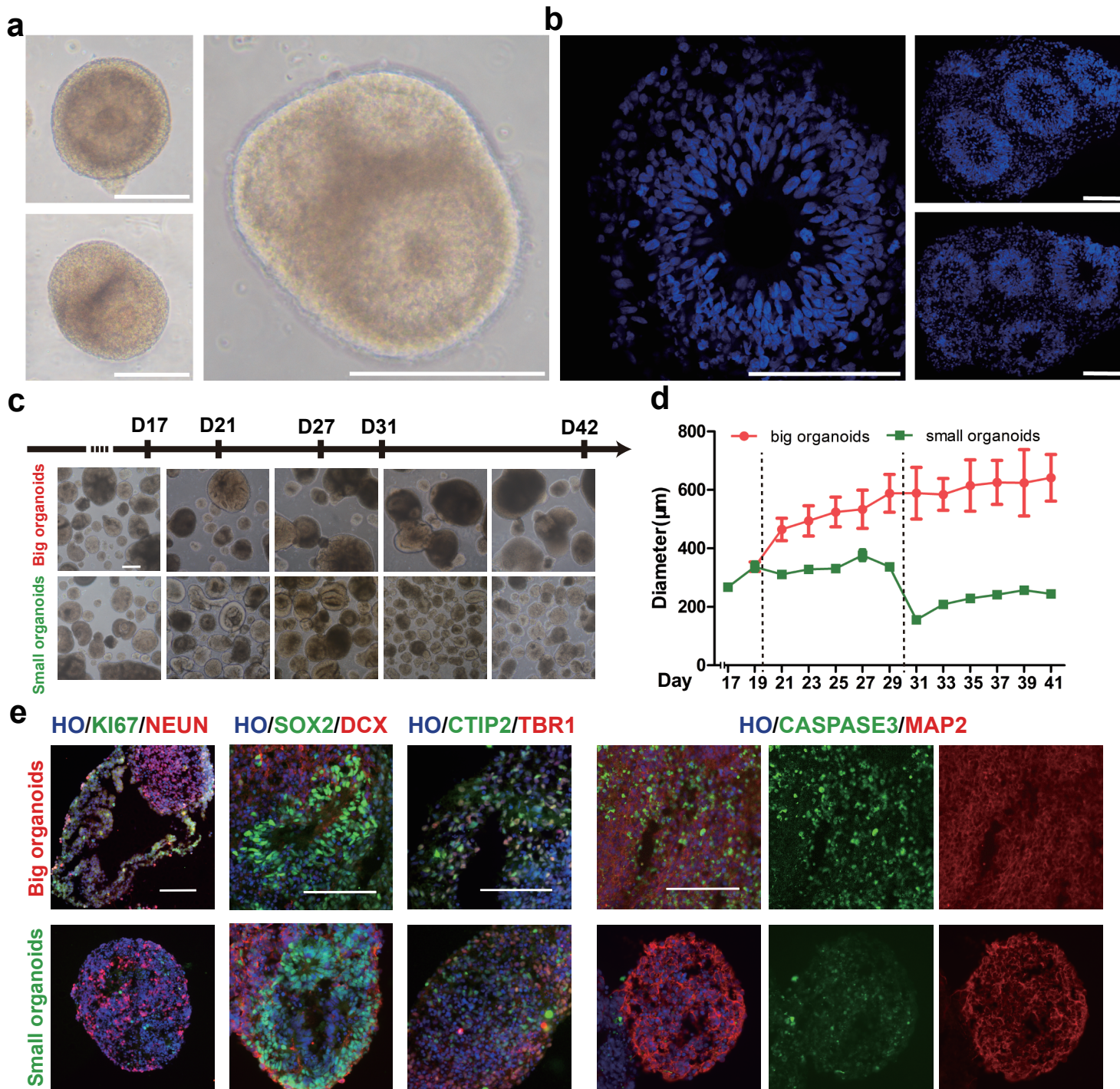

Supplement: Supplementary file 2 — Supplementary Fig. 1 [file 41380_2020_910_MOESM2_ESM.pdf]

## Supplementary Fig. 2

a

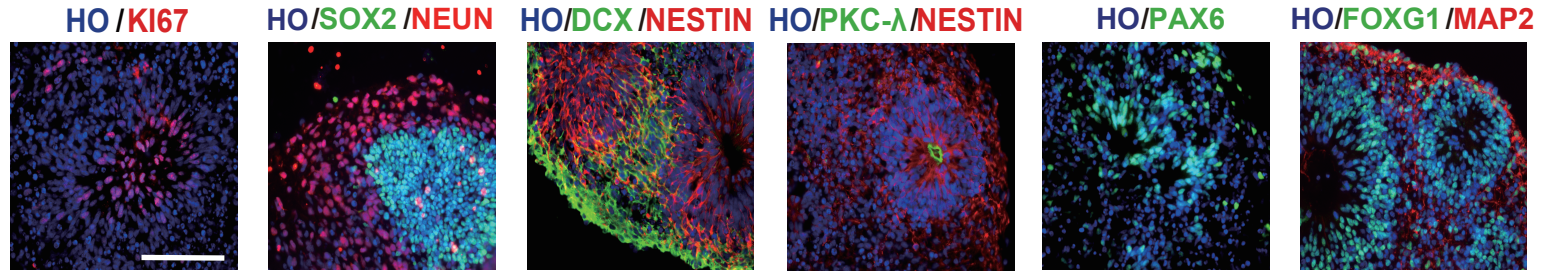

b

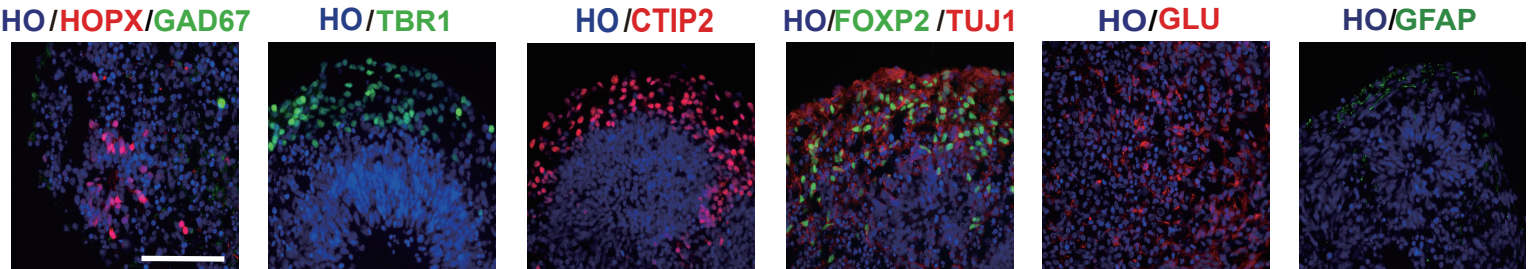

c

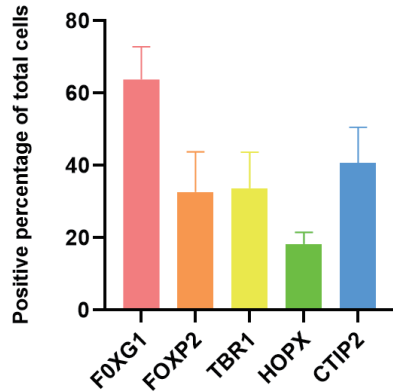

Supplement: Supplementary file 3 — Supplementary Fig. 2 [file 41380_2020_910_MOESM3_ESM.pdf]

Supplementary Fig. 3

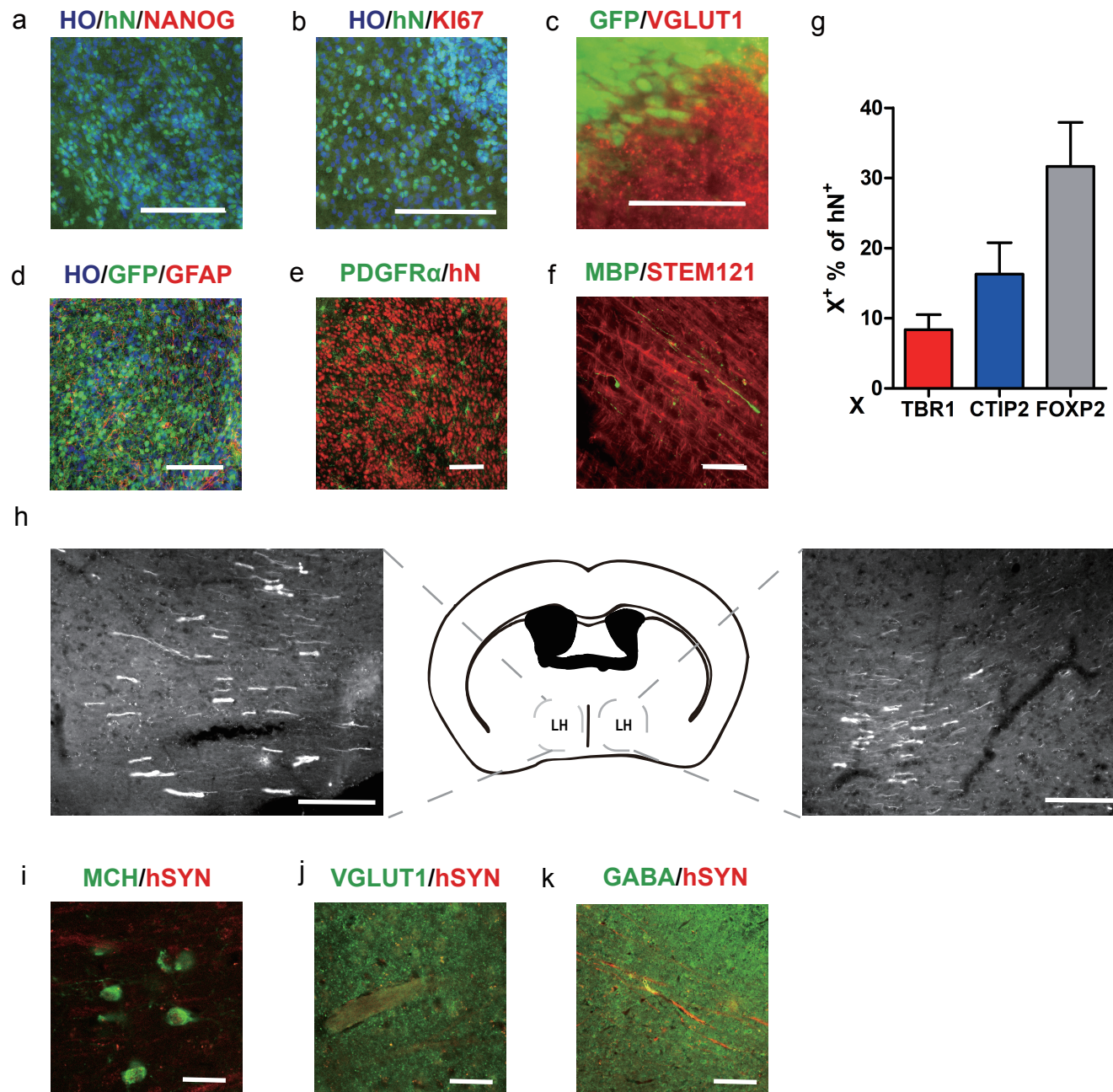

Supplement: Supplementary file 4 — Supplementary Fig. 3 [file 41380_2020_910_MOESM4_ESM.pdf]

# Supplementary Fig. 4

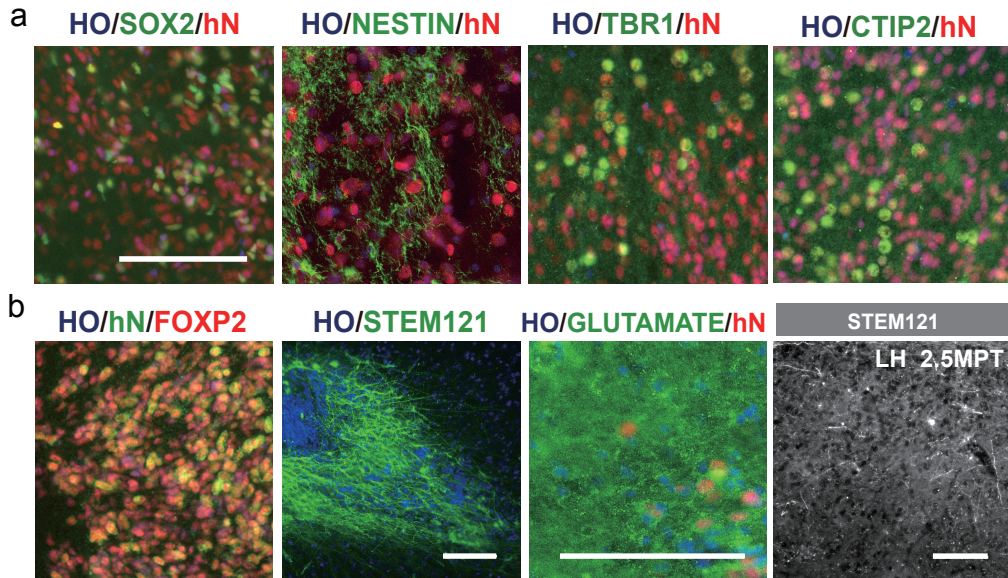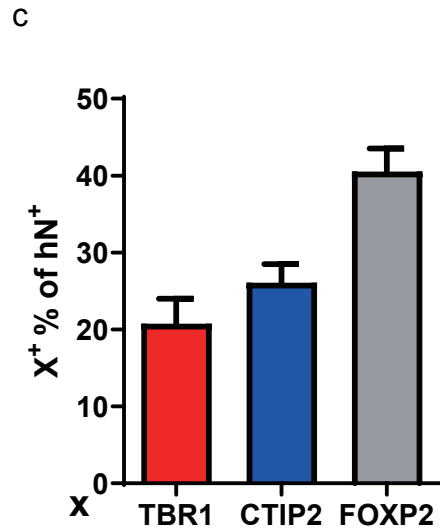

Supplement: Supplementary file 5 — Supplementary Fig. 4 [file 41380_2020_910_MOESM5_ESM.pdf]

# Supplementary Fig. 5

a

Fear acquisition (IMR90-4 line)

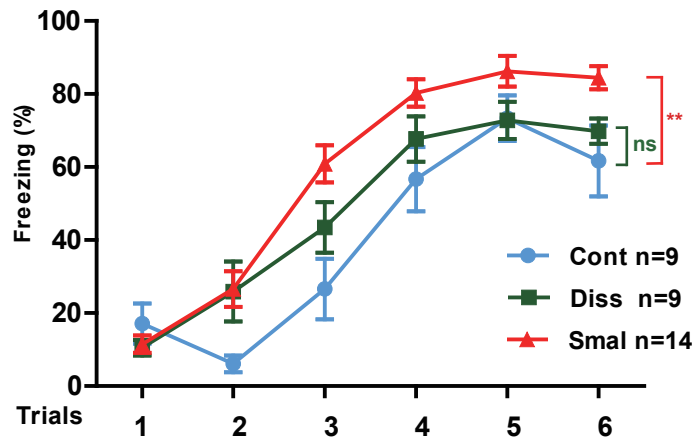

b

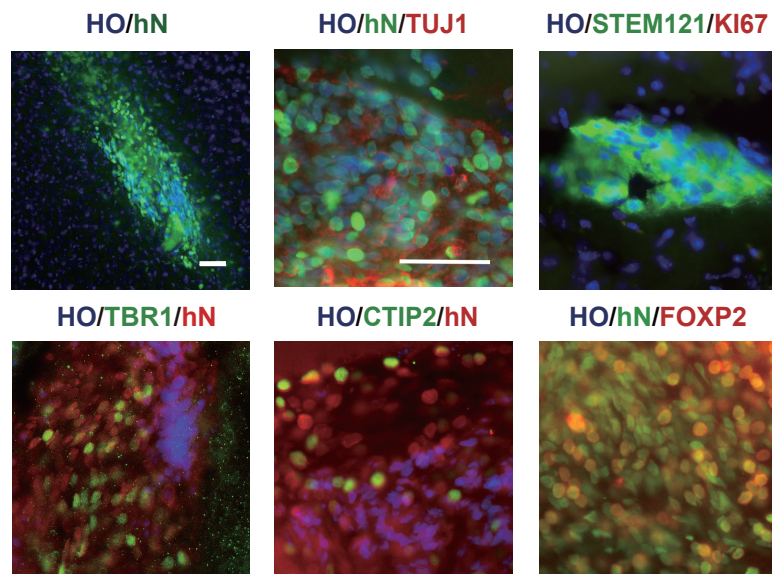

c

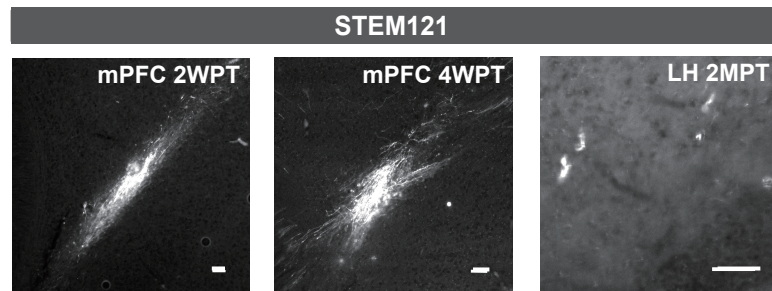

Supplement: Supplementary file 6 — Supplementary Fig. 5 [file 41380_2020_910_MOESM6_ESM.pdf]
