# Supplementary material for: Human cerebral organoids establish subcortical projections in the mouse brain after transplantation
Source: Mol Psychiatry. 2020 Oct 13;26(7):2964–76. doi: 10.1038/s41380-020-00910-4 (PMC8505255; doi:10.1038/s41380-020-00910-4)
Supplement: Supplementary file 7 — Supplementary figure legends [file 41380_2020_910_MOESM7_ESM.docx]

**Supplementary Fig. 1 Differentiation of hPSCs into small organoids**

(a) Under a bright field microscope, the small cerebral organoids exhibited one, two, or three neural tubes.

(b) Immunostaining images showing small cerebral organoids labeled with Hoechst.

(c) Comparison of small cerebral organoids and large cerebral organoids under a bright field microscope.

(d) The diameters of small cerebral organoids and large cerebral organoids during differentiation. The data are shown as the mean ± SEM.

(e) Comparison of immunofluorescence staining of large cerebral organoids and small cerebral organoids at day 40. Big organoids presented a necrotic center. Increased CASPASE3 was detected around the lumens of large organoids, which indicated that large organoids underwent more cell death than small organoids. Small organoids showed higher MAP2 expression than large organoids.

Scale bars: 100 µm.

**Supplemental Fig. 2 Differentiation of small human cerebral organoids from IMR90-4 cells**

(a) Representative immunostaining for the proliferation marker Ki67, ventricular zone marker SOX2, mature neuronal marker NeuN, newborn neuron marker DCX, adherent junction marker PKC-λ, neural stem cell marker Nestin, and dorsal telencephalic progenitor marker PAX6 at day 45.

(b) Images of immunostaining for FOXG1 (the right panel in Supplemental Fig. 2a), HOPX, GAD67, TBR1, CTIP2, FOXP2, TUJ1 and MAP2 in cerebral organoids. The glutamatergic neuronal marker glutamate (GLU) was expressed in organoids derived from the IMR90-4 cell line. However, a low level of glial fibrillary acidic protein (GFAP, human astrocytic marker) was detected.

(c) Quantification of cell populations *in vitro* at day 45 postdifferentiation (n=20 organoids).

Scale bars: 100 μm.

**Supplemental Fig. 3 Immunostaining for human grafts.**

(a) At 1 month posttransplantation, no NANOG^+^ cells were observed among the hN+ cells.

(b) The cell proliferation marker KI67 was rarely observed.

(c) Human grafts contain glutamatergic terminals, as visualized by staining for vesicular glutamate transporter 1 (VGLUT1, a marker of excitatory presynaptic terminals).

(d) A total of 5% of the grafted cells were positive for GFAP.

(e-f) The oligodendrocyte markers PDGF-a and MBP began to be expressed in the grafted cells.

(g) Quantification of CTIP2^+^, TBR1^+^ and FOXP2^+^ cell populations in the hN^+^ graft area (n = 3 mice, mean ± SEM).

(h) One month later, STEM 121^+^ human neurites were detected in the lateral hypothalamus in

coronal sections (n=5 mice).

(i-j) The human neurites were found in the lateral hypothalamus, while the orexin-expressing neuron marker MCH, glutamatergic neuron marker VGLUT1, and GABAergic neuron marker GABA were also expressed in this region.

Scale bars: 100 µm.

**Supplemental Fig. 4 The organoid grafts derived from the IMR90-4 cell line undergo a similar differentiation program *in vivo***

(a) Double immunostaining for the neural progenitor cell marker SOX2, radial glia marker NESTIN, and cortical neuron markers TBR1 and CTIP2 with human nuclei (hN).

(b) The deep cortical layer marker FOXP2 and glutamate (a glutamatergic neuronal marker) were expressed in the grafts. Two and a half months posttransplantation, a high level of STEM121 (human neurite marker) was expressed in the mPFC, and extensive human neuron fibers were detected in LH.

(c) Quantification of CTIP2^+^, TBR1^+^ and FOXP2^+^ cell populations in the hN^+^ graft area (n = 5 mice, mean ± SEM).

Scale bars; 100 µm; the unlabeled scale bars are the same as those in the left panel in Supplemental Fig. 4a.

**Supplemental Fig. 5**

**Comparison of startle fear response among the small organoid group, dissociated neural cell group and control group.**

(a) The startle fear response was increased in the small cerebral organoid (IMR90-4 line) group at 8 weeks after transplantation. Cont: control group, Smal: small organoid group, Diss: dissociated neural cell group. The data are shown as the mean ± SEM.

(b) Double immunostaining for TUJ1, TBR1, CTIP2, FOXP2 with hN and for STEM12 with Ki67 was observed in the dissociated neural cell grafts. Scale bars: 50 µm; the scale bar in the remaining panel is the same as that in the upper middle panel.

(c) The human neurite marker STEM121 was observed at the mPFC 2 and 4 weeks posttransplantation. Few STEM121^+^ fibers were observed in LH in the dissociated neural cell group two months posttransplantation. Scale bars: 50 µm.
